# Photoinduced Metal-Free Surface Initiated ATRP from Hollow Spheres Surface

**DOI:** 10.3390/polym11040599

**Published:** 2019-04-02

**Authors:** Chun-Na Yan, Qian Liu, Lin Xu, Li-Ping Bai, Li-Ping Wang, Guang Li

**Affiliations:** 1 College of Materials Science and Engineering, Liaocheng University, Liaocheng 252059, China; YCN5053@163.com (C.-N.Y.); liuqianbang@163.com (Q.L.); blp9641@163.com (L.-P.B.); 2 College of Materials Science and Engineering, Qingdao University, Qingdao 266071, China; xulinbang@163.com

**Keywords:** hollow spheres, metal-free ATRP, SI-ATRP, 10-phenylphenothiazine (PTH), thermal stability

## Abstract

Well-defined amphiphilic diblock copolymer poly (methyl methacrylate)-*b*-poly (N-isopropylacrylamide) grafted hollow spheres (HS-*g*-PMMA-*b*-PNIPAM) hybrid materials were synthesized via metal-free surface-initiated atom transfer radical polymerization (SI-ATRP). The ATRP initiators α-Bromoisobutyryl bromide (BIBB) were attached onto hollow sphere surfaces through esterification of acyl bromide groups and hydroxyl groups. The synthetic ATRP initiators (HS-Br) were further used for the metal-free SI-ATRP of methyl methacrylate (MMA) and N-isopropyl acrylamide (NIPAM) using 10-phenylphenothiazine (PTH) as the photocatalyst. The molecular weight of the polymers, structure, morphology, and thermal stability of the hybrid materials were characterized via gel permeation chromatography (GPC), X-ray photoelectron spectroscopy (XPS), ^1^H-nuclear magnetic resonance spectroscopy (^1^H NMR), transmission electron microscopy (TEM), Fourier transform infrared spectroscopy (FT-IR), and thermogravimetric analysis (TGA), respectively. The results indicated that the ATRP initiator had been immobilized onto HS surfaces successfully followed by metal-free SI-ATRP of MMA and NIPAM, the Br atom had located at the end of the main PMMA polymer chain, and the polymerization process possessed the characteristic of controlled/“living” polymerization. The thermal stability of the hybrid materials was increased significantly compared to the pure PMMA and PNIPAM.

## 1. Introduction

Surface-initiated radical polymerization is an important synthetic method for the preparation of polymer-inorganic hybrid materials [1,2,3,4,5]. Different “living” radical polymerization methods, such as atom transfer radical polymerization (ATRP) [6,7,8], reversible addition-fragmentation chain transfer (RAFT) [9,10,11], and reverse iodine transfer polymerization (RITP) [12,13,14], have been used to modify inorganic material surfaces with controlled polymer chain length, composition, and architecture via surface-initiated radical polymerization methods. Atom transfer radical polymerization (ATRP) is one of the most versatile and robust methods for synthesis of polymers with controlled molecular weight (MW), molecular weight distribution (MWD), structure, and functionality [15,16]. Surface-initiated ATRP has been successfully used to graft polymers from a variety of solid surfaces of different shapes and materials, such as α-Fe_2_O_3_ rods [17], iron oxide magnetic nanoparticles [18], SBA-15 [19], wood flour [20], and so on. However, ATRP is also practically discouraged due to the disadvantage of using ATRP metal catalysts, such as Cu^+^ and Fe^2+^, which result in the presence of an inevitable metal residue in final products [21,22,23]. Although successful attempts for various low-ppm catalyst ATRP methods have been claimed [24,25,26,27], low levels of catalyst remained in the products, which impeded their use, especially in some electronic or biomedical applications [28,29]. 

In 2014, Treat et al. first reported a metal-free ATRP process, which was mediated by light and catalyzed by an organic-based photoredox catalyst, 10-phenylphenothiazine (PTH) [30]. The new photoinduced metal-free ATRP does not require heavy metal catalysts (such as copper ions and iron ions), so it can avoid the toxicity of metal catalysts. Extensive research has been carried out on photoinduced metal-free ATRP since then. The research group led by Y. Yagci reported a novel synthetic strategy for the syntheses of hyperbranched homo and block copolymers by applying the photoinduced metal-free ATRP [31], and the photoinduced metal-free ATRP activated by highly conjugated electron-rich thienothiophene derivatives was also developed by this group [32]. Wang and co-workers demonstrated metal-free ATRP of biomass-based monomers (e.g., soybean oil, rosin acid, and furfural) under low intensity UV LED light [33]. Matyjaszewski and co-workers further investigated the mechanism of photoinduced metal-free ATRP [34] and expanded the photoredox catalysts using three phenothiazine derivatives as catalysts for photoinduced metal-free ATRP of acrylonitrile [35]. Photoinduced metal-free ATRP was also employed to the surface modification of various materials, such as silica [28], SBA-15 [36], nanodiamond (ND) [37], hydroxyapatite (HAp) nanorods [38], and others.

SiO_2_ hollow spheres (HS) with a large internal void, hollow cavity, small density, and large specific surface area have received much attention owing to their wide potential applications in adsorption and biomacromolecules delivery [39], electrical materials and catalysis [40], optical performance [41], controlled drug-delivery carriers [42], electrical sensing devices [43], and so on [44,45]. Although pure SiO_2_ HS have excellent properties, their applications have been largely restricted due to their poor hydrophobicity and single physical loading ability. Therefore, the surface modification of SiO_2_ HS with different polymers to improve their dispersibility in various solvents and to extend their physicochemical properties is very essential [46,47,48]. 

In the present work, the well-defined HS-*g*-PMMA and HS-*g*-PMMA-*b*-PNIPAM hybrid materials were prepared via metal-free surface-initiated atom transfer radical polymerization (SI-ATRP) for the first time. Firstly, the ATRP initiators α-Bromoisobutyryl bromide (BIBB) were attached onto hollow sphere surfaces through esterification of acyl bromide groups and hydroxyl groups. The synthetic ATRP initiators (HS-Br) could be further used for surface polymerization with methyl methacrylate (MMA) and N-Isopropyl acrylamide (NIPAM) monomer using 10-phenylphenothiazine (PTH) as the photocatalyst. The hybrid materials synthesized through metal-free SI-ATRP will broaden the application prospect of ATRP and HS.

## 2. Materials and Methods

### 2.1. Materials

NaOtBu, phenothiazine, RuPhos Precat, and RuPhos were purchased from Sigma-Aldrich (Shanghai, China). α-Bromoisobutyryl bromide (BIBB) was acquired from Shanghai Aladdin Chemical Reagent Co. Ltd (Shanghai, China). Methyl methacrylate (MMA), N-Isopropyl acrylamide (NIPAM), and Dimethylacetamide (DMA) were purchased from Shanghai Sinopharm Chemical Reagent Co. Ltd (Shanghai, China). 10-Phenylphenothiazine (PTH) and the hollow spheres were synthesized according to previously described literature, respectively [30,49]. The ^1^HNMR of PTH can be found in Figure S1. All other chemical reagents were analytical grade and used without further purification.

### 2.2. Instrumental Characterization

The molecular weights and polydispersities (PDI) of the polymers were measured at 30 °C on a gel permeation chromatographer (GPC) (Waters 1515, Milford, MA, USA) equipped with a 2414 refractive detector. Tetrahydrofuran (THF) was used as an eluent at a flow rate of 0.6 mL/min^−1^. Transmission electron microscope (TEM) images were obtained using a JEOL JEM-1400 (JEOL, Tokyo, Japan) transmission electron microscope. The diameter of wall thickness and the average diameter of the hollow spheres were calculated using Intel Integrated Performance Primitives (ipp). ^1^H NMR spectra were recorded on a 400 MHz (Varian Mercury Plus 400, Palo Alto, CA, USA) nuclear magnetic resonance instrument, using CDCl_3_ as solvent and tetramethylsilane (TMS) as the internal reference. The structures of samples were characterized by Nicolet-100 Fourier (Thermo Fisher Scientific, MA, USA) transform infrared spectroscopy (FT-IR) from 400 cm^−1^ to 4000 cm^−1^ using the KBr pellet method. X-ray photoelectron spectroscopy (XPS) was performed on an Escalab 250xi (Thermo Fisher Scientific, MA, USA) spectrometer equipped with an Al Kα X-ray source (hv = 1486.6 eV), with a take-off angle of 0 degrees. The thermal decomposition behaviors of the materials were recorded on an STA 449C simultaneous DSC-TGA (Netzsch Instruments, Selb, Germany) thermal gravimetric analyzer (TGA) with a heating rate of 10 °C/min^−1^ in nitrogen atmosphere. 

### 2.3. Synthesis of HS-Br

HS-Br was prepared via the esterification of acyl bromide groups in BIBB and hydroxyl groups in HS. The following procedure was adopted: 2 g SiO_2_ HS and 15 mL dichloromethane were added to a 100 mL reactor equipped with a magnetic stirrer. The mixture was stirring at room temperature for 10 min. Subsequently, the solution of BIBB (1.2 mL, 0.01 mol) dissolved in 5 mL dichloromethane was dropped slowly into the round-bottom flask by a constant pressure liquid funnel. After reacting for 12 h at room temperature, the crude HS-Br was separated by centrifugation and washed completely by Soxhlet extraction with THF. The obtained HS-Br initiator was dried at 30 °C in vacuum. Elem. Anal. Calcd. (%): C, 2.37; H, 3.4; Br, 0.473 mmol g^−1^ (calculated according to the C content) (Table 1).

### 2.4. Synthesis of HS-g-PMMA

HS-*g*-PMMA was been synthesized through a new method of metal-free ATRP. In brief, HS-Br (0.15 g, 0.071 mmol Br groups), Methyl methacrylate (MMA) (1.6 mL, 15 mmol), PTH (0.0042 g, 0.015 mmol), Ethyl 2-bromoisobutyrate (5.5 μL, 0.0375 mmol), and 1 mL DMA were mixed in a 10 mL quartz tube with a magnetic stirrer. The reaction mixture was stirred for 10 min in the dark at room temperature. When the reaction system was uniformly dispersed, the reaction system was irradiated under an ultraviolet lamp with 365 nm in the dark at room temperature; after reacting for 5 h, the reaction mixture was separated by centrifugation. The obtained solids (crude HS-*g*-PMMA) were precipitated in methanol respectively and dried at 40 °C in vacuum. Afterwards, the HS-*g*-PMMA hybrid material was obtained by Soxhlet’s extraction with THF for 12 h to remove the ungrafted PMMA completely and dried at 40 °C in vacuum. The solution after centrifugation was also precipitated in methanol and dried to afford PMMA. Elem. Anal. Calcd. (%): C, 6.38; H, 1.34; PMMA, 0.668 mmol g^−1^ (calculated according to the C content) (Table 1).

### 2.5. Synthesis of HS-g-PMMA-b-PNIPAM

**Scheme 1:**
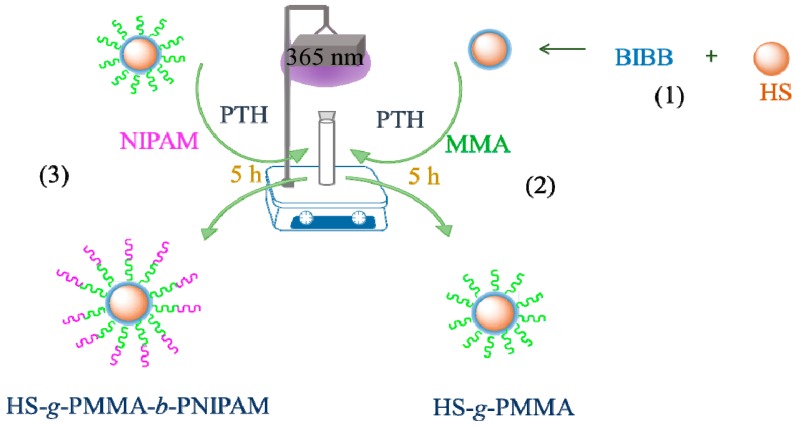
The synthetic routes of HS-*g*-PMMA and HS-*g*-PMMA-*b*-PNIPAM.

HS-*g*-PMMA-*b*-PNIPAM was synthesized through a new method of metal-free ATRP using HS-*g*-PMMA as the macroinitiaor. In brief, 0.1 g HS-*g*-PMMA, NIPAM (0.42 g, 3.75 mmol), PTH (0.001 g, 0.00375 mmol), Ethyl 2-bromoisobutyrate (1.67 μL, 0.0125 mmol), and 1 mL DMA were mixed in a 10 mL quartz tube with a magnetic stirrer. The reaction mixture was stirred for 10 min in the dark at room temperature. When the reaction system was uniformly dispersed, the reaction system was irradiated under an ultraviolet lamp with 365 nm in the dark at room temperature; after reacting for 5 h, the reaction mixture was separated by centrifugation. The obtained solids (crude HS-*g*-PMMA-*b*-PNIPAM) were precipitated in methanol and dried at 40 °C in vacuum. Then, the crude HS-*g*-PMMA-*b*-PNIPAM was extracted with THF to remove the ungrafted polymers completely. The solution after centrifugation was precipitated in cold ether and resulted in PNIPAM. The detailed reaction process is listed in Scheme 1. Elem. Anal. Calcd. (%): N, 1.24; C, 10.01; H, 1.81; PNIPAM, 0.504 mmol g^−1^ (calculated according to the C content) (Table 1), so the molar ratio of PMMA and PNIPAM for HS-*g*-PMMA-*b*-PNIPAM is 1.32:1.

## 3. Results and Discussion

### 3.1. Structure Analysis

**Figure 1:**
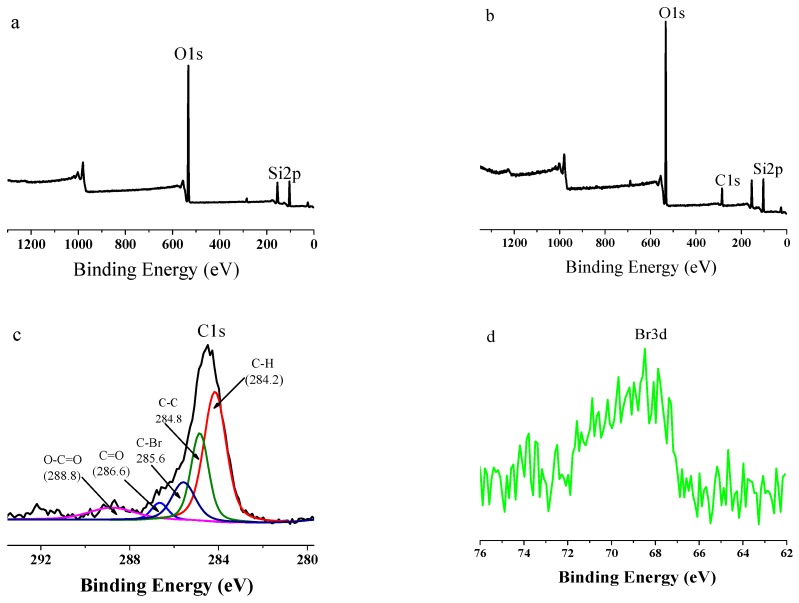
X-ray photoelectron spectroscopy (XPS) spectra of HS (**a**) and HS-Br (**b**–**d**): Survey scan curves range from 0 to 1350 eV (**b**), the C1s core-level spectra (**c**) and Br3d region (**d**).

HS-*g*-PMMA-*b*-PNIPAM was synthesized by metal-free surface-initiated ATRP (SI-ATRP). To prepare the copolymer brushes grafted from the HS surface via SI-ATRP, the immobilization of the ATRP initiator onto HS surfaces is necessary. The chemical compositions of HS and HS-Br were detected by XPS analysis. As shown in Figure 1, the surface of a HS is mainly composed by Si and O elements. Compared with crude HS, the HS-Br surfaces clearly show new C and Br elements. The peaks of C1s (284.5 eV) and Br3d (68.4 eV) are ascribed to the C and Br of BIBB on the HS surface. The C1s core-level spectra of the HS-Br surfaces (Figure 1c) could be curve-fitted into five peak components, which were attributable to the O–C=O, C=O, C–Br, C–C, and C–H species of HS-Br, respectively. The observed signals for Si_2p_ (102.1 eV) are attributed to exposed Si element of HS. The C, Br, and Si signals suggested that the ATRP initiator has been successfully immobilized onto HS surfaces, which provides the necessary conditions for the following metal-free surface-initiated ATRP. The element compositions (atomic %) of C and Br in HS-Br based on XPS analysis are listed in Table S1.

**Figure 2:**
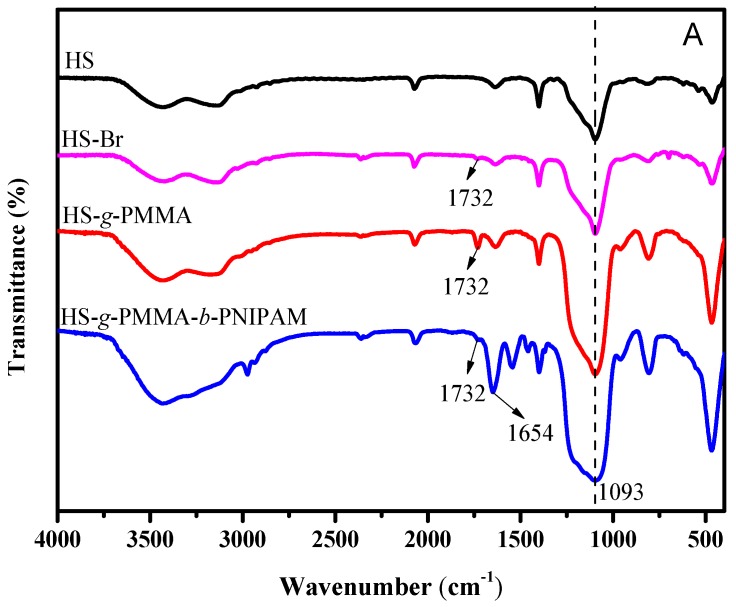

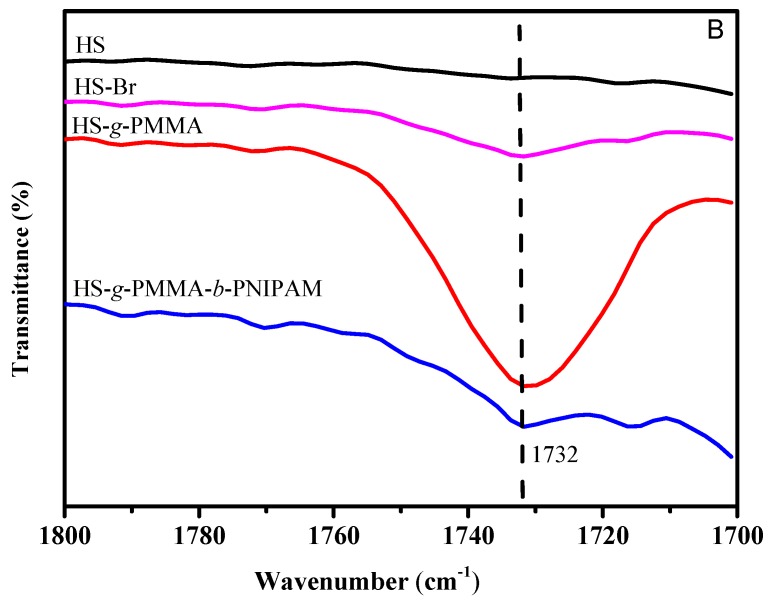
Fourier transform infrared spectroscopy (FT-IR) spectra (**A**) and magnified FT-IR spectra in the region 1700–1800 cm^−1^ (**B**) of hollow spheres, HS-Br, HS-*g*-PMMA, and HS-*g*-PMMA-*b*-PNIPAM.

Figure 2 shows the FT-IR spectra of the HS, HS-Br, HS-*g*-PMMA, and HS-*g*-PMMA-*b*-PNIPAM. The peaks at 1093 cm^−1^ are attributed to the stretching vibration absorption of Si-O-Si of HS. Compared with crude HS, the FT-IR spectra of HS-Br clearly shows a new characteristic peak at 1732 cm^−1^ which belongs to the C=O stretching vibration of BiBB (Figure 2A,B), indicating the successful esterification of acyl bromide groups and hydroxyl groups. After metal-free SI-ATRP, the enhancive peak at 1732 cm^−1^ belongs to the C=O stretching vibration which is the result of the C=O overlap of BiBB and PMMA. As for the HS-*g*-PMMA-*b*-PNIPAM, the absorption peak at 1654 cm^−1^ accords with the –CO–N– stretching vibration of PNIPAM, and the decreased peak at 1732 cm^−1^ resulted from the C=O stretching vibration of PMMA, which indicated the successful grafting of PMMA and PNIPAM onto HS surfaces.

**Figure 3:**
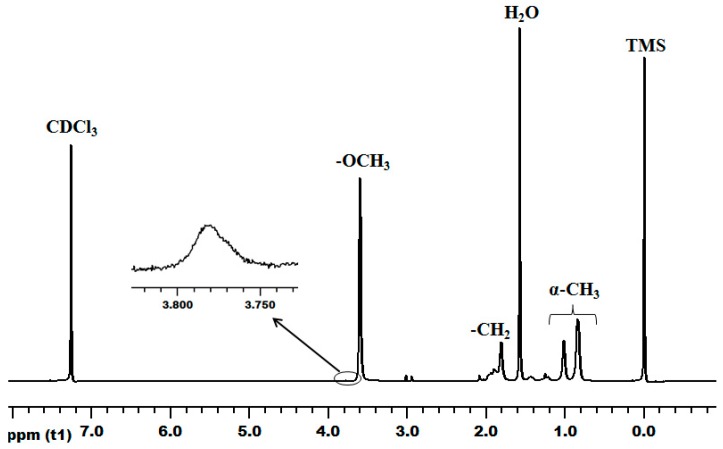
^1^H-nuclear magnetic resonance spectroscopy (^1^H NMR) spectrum of PMMA formed in solution.

The ^1^H NMR spectrum of PMMA formed in solution also gives support to the metal-free SI-ATRP mechanism (Figure 3). Except for the peak of NMR internal reference (TMS) at 0.00 ppm and solvent (CDCl_3_) at 7.27 ppm, the signals at around 1.81 ppm and 0.80–1.02 ppm are ascribed to the protons of methylene and methyl to the repeat unit in the main polymer chain. The peak at 3.78 ppm is attributed to the methyl ester group (–OCH_3_) at the chain end in PMMA, as mentioned by Zhang [19], which deviated from the chemical shift (3.60 ppm) of the other –OCH_3_ due to the electron attracting function of the Br atom at the chain end. The ^1^H NMR spectrum result indicated that the Br atom was located at the end of the main PMMA polymer chain. 

**Figure 4:**
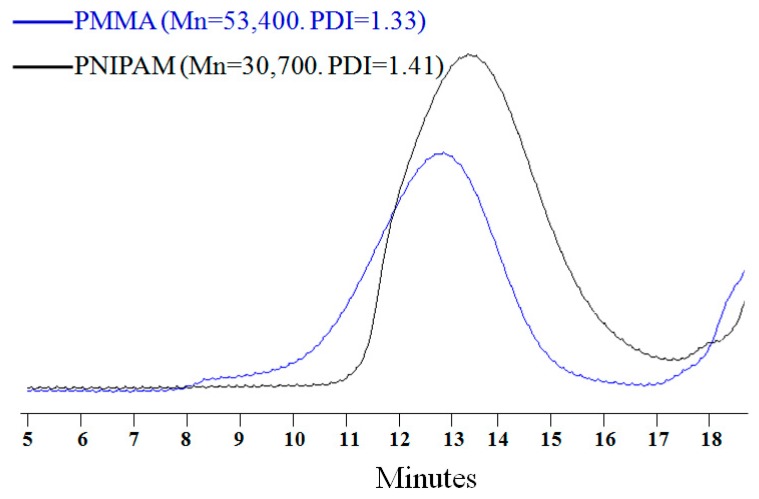
Gel permeation chromatography (GPC) traces of PMMA and PNIPAM.

Figure 4 show the GPC traces of PMMA and PNIPAM formed in solution. The molecular weight (Mn) values reached 53,400 and 30,700 g/mol, respectively, and the molecular weight distributions (Mw/Mn) were narrow (1.33 and 1.41, respectively), which indicated that the polymerization process possesses the characteristic of controlled/“living” polymerization.

### 3.2. Morphology Analysis

**Figure 5:**
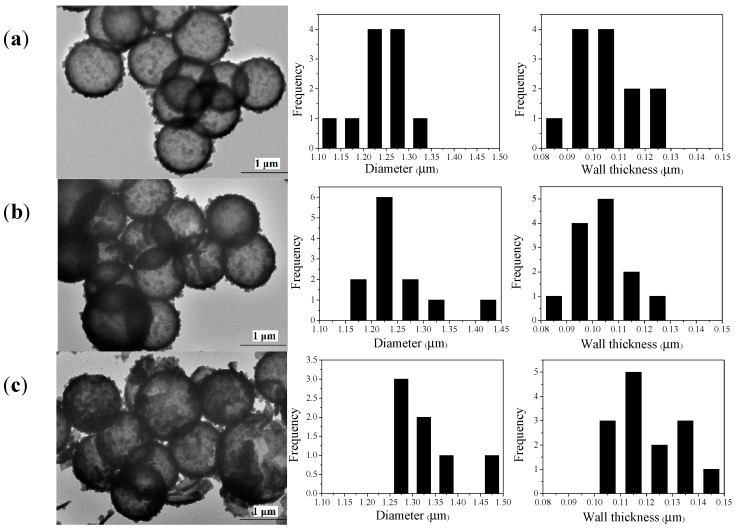
Transmission electron microscope (TEM) images of (**a**) hollow spheres, (**b**) HS-*g*-PMMA, and (**c**) HS-*g*-PMMA-*b*-PNIPAM.

In order to obtain more information on the effects of polymer grafting onto the microstructures of hollow spheres, TEM was used to investigate the surface topography in detail. Figure 5 shows the TEM images of hollow spheres, HS-*g*-PMMA, and HS-*g*-PMMA-*b*-PNIPAM. The hollow nature of the hollow spheres was observed by the contrast in TEM observations, as shown in Figure 5, and it can be observed that the diameter of the wall thickness exhibits a tendency to increase with process of grafting polymerization. The average diameter of the hollow spheres mainly centered at 1.2–1.3 μm, and the average wall thickness mainly focused on 90–110 nm before grafting polymerization, as indicated by the size distribution histogram (Figure 5a). After grafting polymerization (Figure 5b,c), a small number of hollow spheres were broken (Figure 5c), and the average wall thickness of the hollow spheres increased from 100 to 105 and 115 nm, respectively, owing to the grafting polymerization of MMA and PNIPAM on the surfaces of hollow spheres.

### 3.3. TGA Analysis

**Figure 6:**
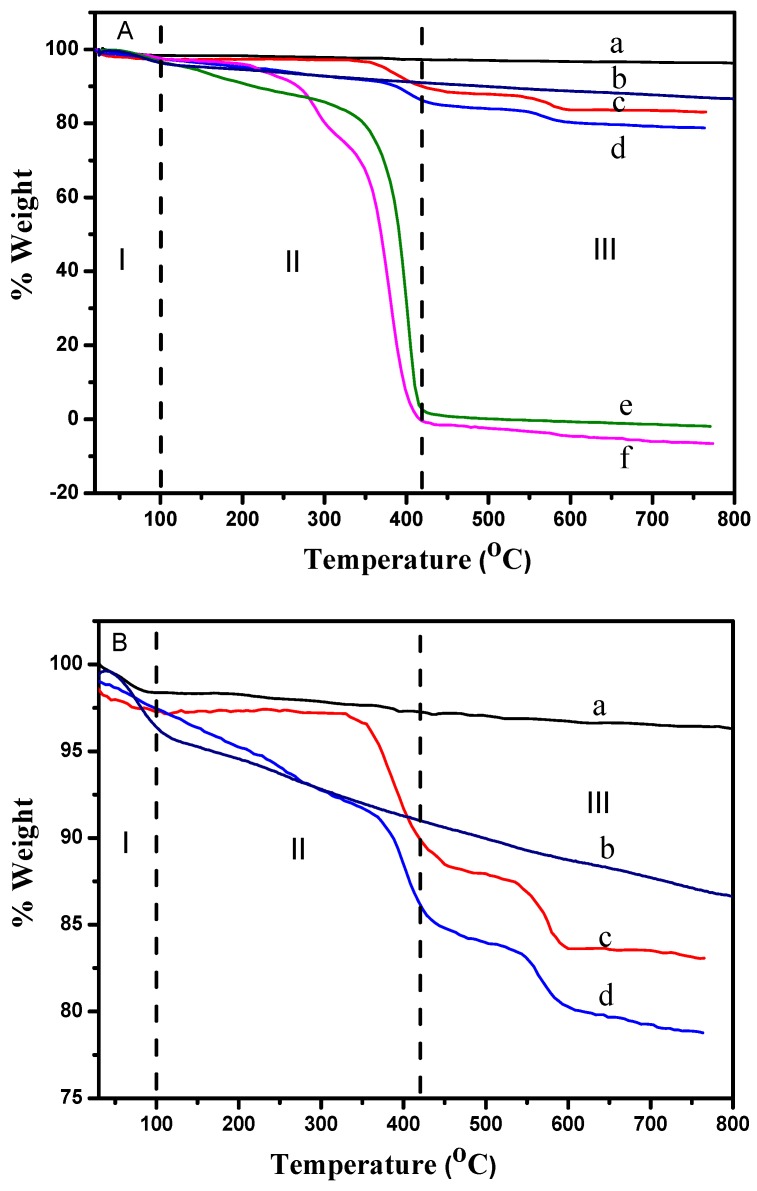
(**A**) TG curves of (a) hollow spheres, (b) HS-Br, (c) HS-*g*-PMMA, (d) HS-*g*-PMMA-*b*-PNIPAM, (e) pure PMMA and (f) pure PNIPAM. (**B**) Magnified TG curves of (a) hollow spheres, (b) HS-Br, (c) HS-*g*-PMMA, and (d) HS-*g*-PMMA-*b*-PNIPAM.

The TGA was used to determine the grafting percentage of polymers on the surface of hollow spheres and investigate the thermostability of hollow sphere samples. Figure 6 shows the TGA curves of hollow spheres, HS-*g*-PMMA, and HS-*g*-PMMA-*b*-PNIPAM with temperature increasing from room temperature to 800 °C. The weight loss experienced by the hollow spheres, HS-*g*-PMMA, and HS-*g*-PMMA-*b*-PNIPAM before 100 °C is attributed to the loss of adsorbed water in the samples (zone I). The second significant weight loss was observed in the range of 100–420 °C (zone II), which is due to the thermal decomposition of the organic content in PMMA and PNIPAM (the thermal decomposition temperature of pure PMMA and pure PNIPAM is 420 °C, Figure 6e,f). The third weight loss zone is associated with the inorganic salts remaining in the hollow spheres (zone III). The pure hollow spheres show no obvious decomposition (3.7%) between room temperature and 800 °C. Therefore, the grafting percentage of polymers on the surfaces of the hollow spheres can be obtained via the weight loss of zone II. The organic content calculated for HS-*g*-PMMA is 7.87%. Compared with the HS-*g*-PMMA, the calculated organic content of PMMA-*b*-PNIPAM increased to 11.49%. That is, the grafted PNIPAM on hollow spheres surface is about 3.62%. This indicated that both PMMA and PNIPAM have successfully grafted onto the hollow sphere surfaces. 

### 3.4. Dispersibility in Solvents

**Figure 7:**
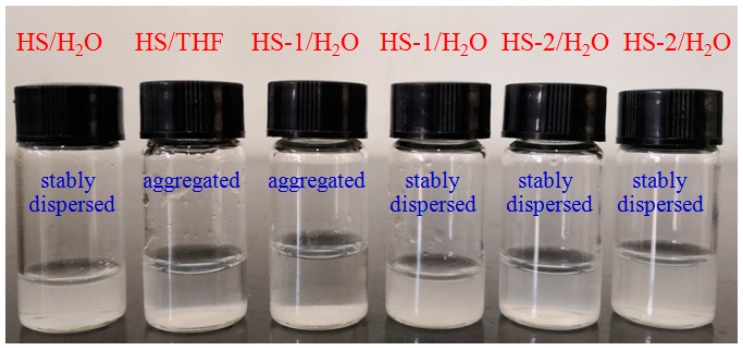
Dispersed/aggregated state of the hollow spheres samples in H_2_O/THF, respectively.

Figure 7 presents the images of the dispersions of hollow spheres samples in inorganic (H_2_O) and organic solvent (THF). The pure HS are dispersed in water and aggregated in THF; the grafting of PMMA onto HS surfaces (HS-1 (HS-*g*-PMMA)) resulted in the complete opposite dispersibility. They are aggregated in the aqueous phase but readily dispersed in THF. These results show that the grafted PMMA contributed to the surface hydrophobization of the hollow spheres. The surface modification of HS with PMMA-*b*-PNIPAM (HS-2 (HS-*g*-PMMA-*b*-PNIPAM)) alters the dispersion again. The PNIPAM chains were shown to contribute to increasing the surface amphiphilicity of HS.

## 4. Conclusions

In summary, we reported a facile synthesis of well-defined amphiphilic diblock copolymer grafted hollow spheres via metal-free SI-ATRP. The ATRP initiators α-Bromoisobutyryl bromide (BIBB) were attached onto the surfaces of hollow spheres to initiate metal-free SI-ATRP of MMA and NIPAM. It was found that the BIBB had been immobilized onto HS surfaces successfully. Characterization with FT-IR, ^1^HNMR, TEM, and TGA verified the success of the metal-free SI-ATRP of MMA, and the Br atom had located at the end of the main PMMA polymer chain, which gives HS-*g*-PMMA the ability to extend the chains by sequential addition of NIPAM. Furthermore, the molecular weight distributions (Mw/Mn) of PMMA and PNIPAM formed in solution is narrow (1.33 and 1.41, respectively), which indicated that the polymerizations process possesses the characteristic of controlled/“living” polymerization.

## Figures and Tables

**Table 1:** Elemental analysis of hollow spheres (HS), HS-Br, HS-1 (HS-*g*-PMMA), HS-2 (HS-*g*-PMMA-*b*-PNIPAM).

	Element/%	N	C	H
Sample				
HS		0	0.10	0.88
HS-Br		0	2.37	3.40
HS-1 (HS-*g*-PMMA)		0	6.38	1.34
HS-2 (HS-*g*-PMMA-*b*-PNIPAM)		1.24	10.01	1.81

## Supplementary Materials

The following are available online at https://www.mdpi.com/2073-4360/11/4/599/s1, Figure S1: ^1^H NMR spectrum of 10-phenylphenothiazine (PTH), Table S1: Element compositions (atomic %) of HS-Br based on XPS analysis.
