# Supplementary material for: Photoinduced Metal-Free Surface Initiated ATRP from Hollow Spheres Surface
Source: Polymers (Basel). 2019 Apr 2;11(4):599. doi: 10.3390/polym11040599 (PMC6523302; doi:10.3390/polym11040599)
Supplement: Supplementary file 1 [file polymers-11-00599-s001.pdf]

polymers

MDPI

Article- Supporting information

Photoinduced Metal-Free Surface Initiated ATRP from Hollow Spheres Surface

Chun-Na Yan [1,†], Qian Liu [1,†], Lin Xu [2], Li-Ping Bai [1], Li-Ping Wang [1,*] and Guang Li [1,*]

[1] College of Materials Science and Engineering, Liaocheng University, Liaocheng 252059, China; YCN5053@163.com (C.-N.Y.); liuqianbang@163.com (Q.L.); blp9641@163.com (L.-P.B.)

[2] College of Materials Science and Engineering, Qingdao University, Qingdao 266071, China; xulinbang@163.com

* Correspondence: wangliping5@163.com (L.-P.W.); lglzsd@126.com (G.L.), Tel.: +86-635-8230-919 (L.-P.W.)

† C.-N.Y. and Q.L. contributed equally.

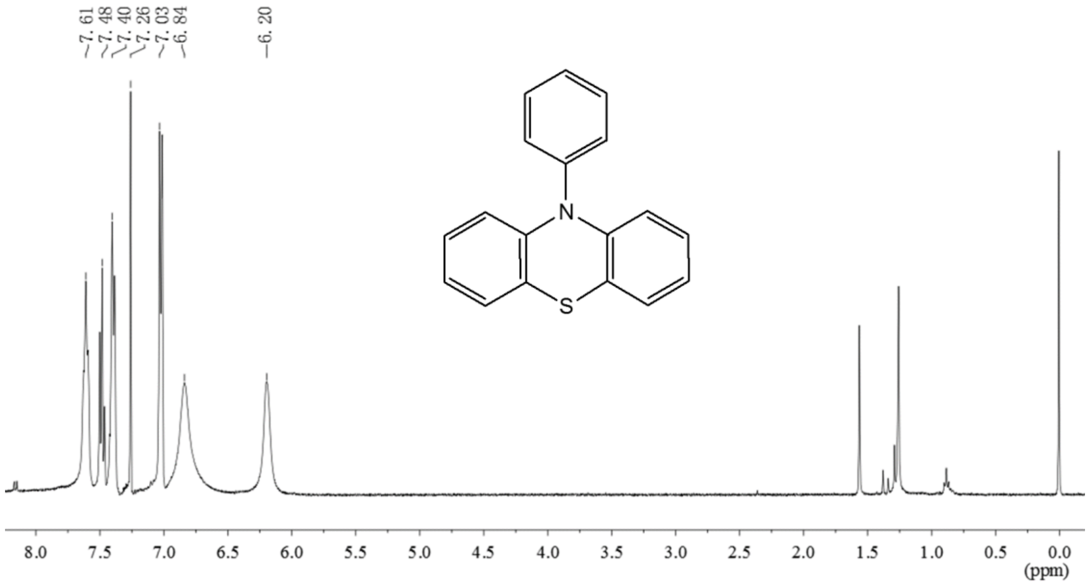

^{1}H NMR (600 MHz, CDCl3) δ: 7.61 (t, J = 8 Hz, 2H), 7.48 (t, J = 8 Hz, 1H), 7.40 (d, J = 7 Hz, 2H), 7.03 (d, J = SI 4 8 Hz, 2H), 6.84-6.79 (m, 4 H), 6.20 (d, J = 8 Hz, 2 H) ppm.

Figure S1. ^{1}H NMR spectrum of 10-phenylphenothiazine (PTH).

Table S1. Element compositions (atomic %) of HS-Br based on XPS analysis.

Sample	C1s	Br3d
HS-Br	98.21	1.79
